# G-Protein-Coupled Receptors: Next Generation Therapeutic Targets in Head and Neck Cancer?

**DOI:** 10.3390/toxins7082959

**Published:** 2015-08-05

**Authors:** Takeharu Kanazawa, Kiyoshi Misawa, Yuki Misawa, Takayuki Uehara, Hirofumi Fukushima, Gen Kusaka, Mikiko Maruta, Thomas E. Carey

**Affiliations:** 1 Department of Otolaryngology-Head and Neck Surgery, Jichi Medical University, Shimotsuke 329-0498, Japan; E-Mail: maruta@jichi.ac.jp; 2 Laboratory of Head and Neck Center Biology, Department of Otolaryngology, Head and Neck Surgery, the University of Michigan, Ann Arbor, MI 48109, USA; E-Mails: kiyoshim@hama-med.ac.jp (K.M.); mswyuki8@hama-med.ac.jp (Y.M.); careyte@med.umich.edu (T.E.C.); 3 Department of Otolaryngology/Head and Neck Surgery, Hamamatsu University School of Medicine, Hamamatsu 431-319, Japan; 4 Department of Otorhinolaryngology, Head and Neck Surgery, Graduate School of Medicine, University of the Ryukyus, Nishihara 903-0215, Japan; E-Mail: yataikyue@hotmail.co.jp; 5 Department of Head and Neck, Cancer Institute Hospital of Japanese Foundation for Cancer Research, Tokyo 135-8550, Japan; E-Mail: hfukusima@jfcr.or.jp; 6 Department of Neurosurgery, Jichi Medical University Saitama Medical Center, Saitama 330-8503, Japan; E-Mail: gkusaka@omiya.jichi.ac.jp

**Keywords:** head and neck neoplasm, biomarker, treatment, molecular targeted therapy

## Abstract

Therapeutic outcome in head and neck squamous cell carcinoma (HNSCC) is poor in most advanced cases. To improve therapeutic efficiency, novel therapeutic targets and prognostic factors must be discovered. Our studies have identified several G protein-coupled receptors (GPCRs) as promising candidates. Significant epigenetic silencing of GPCR expression occurs in HNSCC compared with normal tissue, and is significantly correlated with clinical behavior. Together with the finding that GPCR activity can suppress tumor cell growth, this indicates that GPCR expression has potential utility as a prognostic factor. In this review, we discuss the roles that galanin receptor type 1 (GALR1) and type 2 (GALR2), tachykinin receptor type 1 (TACR1), and somatostatin receptor type 1 (SST1) play in HNSCC. GALR1 inhibits proliferation of HNSCC cells though ERK1/2-mediated effects on cell cycle control proteins such as p27, p57, and cyclin D1, whereas GALR2 inhibits cell proliferation and induces apoptosis in HNSCC cells. Hypermethylation of *GALR1*, *GALR2*, *TACR1*, and *SST1* is associated with significantly reduced disease-free survival and a higher recurrence rate. Although their overall activities varies, each of these GPCRs has value as both a prognostic factor and a therapeutic target. These data indicate that further study of GPCRs is a promising strategy that will enrich pharmacogenomics and prognostic research in HNSCC.

## 1. Introduction

Head and neck carcinomas are defined as carcinomas of head and neck regions including pharynx, larynx, the tongue, oral cavity, nasal cavity and paranasal cavity. They are usually characterized histopathologically as squamous cell carcinomas. Current standard treatments for head and neck squamous cell carcinomas (HNSCC) are aggressive and multimodal treatments including surgery, radiotherapy, and chemotherapy. Despite these aggressive treatments, long-term survival rates are poor and remain between 40% and 50% [1,2,3]. Surgical intervention is challenging in HNSCC cases, as there is a limited surgical margin; this is because tumors are located close to vital organs such as those in the central nervous system, carotid artery, trachea, and esophagus. Furthermore, surgery can lead to serious functional disorders such as dysphagia, or mastication and communication disorder following removal of the tongue, pharynx, and larynx. Radiotherapy is also an effective treatment of early stage HNSCC, but has limited utility in advanced stages. Chemotherapy shows great promise for future treatment regimens, but the optimal regimens remain to be determined. Additionally, most of agents used in HNSCC treatment are cytotoxic and elicit serious side effects [4,5].

The molecular targeted agent Cetuximab is a chimeric monoclonal antibody designed as inhibitor of epidermal growth factor receptor (EGFR) function [6]. Following an initial wave of optimism for its use to treat advanced HNSCC, it was found that this biologic agent was no more effective than other treatments, and in some cases was associated with new side effects [6]. Furthermore, intrinsic and acquired resistance to this agent is a common clinical outcome [6,7].

To improve the survival rate of HNSCC patients, there is a requirement for novel treatment strategies that are less toxic, and that can improve survival in the long term. In turn, this creates the need for development of new drugs and identification of novel biomarkers.

The sensitivity of HNSCC to radiotherapy/chemotherapy is case-specific due to its complex etiology; disease risk is increased by extrinsic factors such as smoking, alcohol and virus infection, which induce factor-dependent genetic alterations [8,9]. With regard to viral infection, human papilloma virus (HPV) infection is an established biomarker to predict responsiveness to radiotherapy and chemotherapy [8]. Indeed, HPV-associated HNSCCs are more sensitive to radiotherapy and chemotherapy than smoking-associated HNSCCs, and HPV infection can therefore be used as a prognostic biomarker [8]. However, HPV-positive HNSCC cases are rare [10], and thus additional biomarkers should be identified to help stratify patients for treatment.

G protein-coupled receptors (GPCRs) modulate the manifold intracellular signaling pathways and can elicit cytostatic and cytotoxic effects, which include apoptosis and cell cycle arrest [11]. Furthermore, epigenetic repression of GPCR expression is closely related to prognosis and/or the response to chemotherapy.

In light of this, the role of GPCRs in HNSCC and their clinical relevance to the disease have been extensively explored [12,13]. In this review, we discuss results of studies on several GPCRs, and discuss the future direction of GPCR-focused studies in HNSCC.

## 2. Galanin and Galanin Receptor Type 1 (GALR1)

### 2.1. The GALR1 Signaling Pathway

GALR1 is one of three GPCRs for a neuropeptide, galanin, encoded by the *GALR1* gene that is widely expressed in the brain, spinal cord, gut and so on. Previous studies in pharmacology demonstrated that stimulation of GALR1 inhibits forskolin-stimulated cAMP production, and this inhibition was observed as a pertussis toxin (PTX)-sensitive manner in transfected cell lines [14,15]. Furthermore, GALR1 activates G protein-regulated inwardly rectifying K^+^ (GIRK) channels [16] and mitogen-activated protein kinase (MAPK) in a protein kinase C (PKC)-independent manner [15]. A critical question is whether galanin and GALR1 can activate MAPK activation in cancer cells, because MAPK is a significant target in cancer therapy [17]. There are conflicting results from studies of the GALR1 signaling pathway with regard to this issue. For example, galanin stimulated extracellular-regulated protein kinase (ERK) activation in 293T cells overexpressing GALR1 [18]. However, in laryngeal carcinoma cell lines, an anti-GALR1 antibody induced ERK activation, suggesting that GALR1 is a negative regulator of ERK [18]. These disparate responses suggest that the results of GPCR activation for the ERK pathway are context-dependent [19].

### 2.2. GALR1 Function in HNSCC

**Figure 1:**
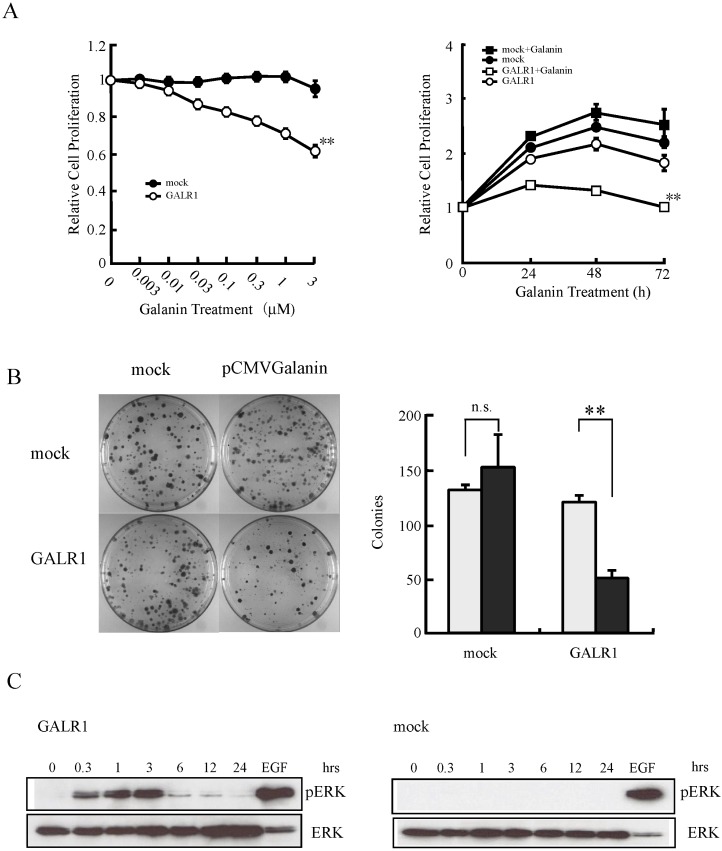
Effect of galanin stimulation on galanin receptor type 1 (*GALR1*)-transfected head and neck squamous cell carcinoma (HNSCC) cells. (**A**) Relative cell proliferation after galanin stimulation. *GALR1* transfected cells were cultured with various concentrations of galanin for 24 h (left) or 1 μM galanin for 24 h, 48 h and 78 h (right). Cell proliferation was significantly inhibited in a concentration and time-dependent manner (** *p* < 0.01); (**B**) Inhibition potential of colony formation by galanin and GALR1. Significant inhibition of colony formation was found in the GALR1-transfected HNSCC cells (** *p* < 0.01); n.s., no significant difference; (**C**) Galanin stimulation induced marked and prolonged extracellular-regulated protein kinase (ERK)1/2 activation in *GALR1*-transfected HNSCC cells. Figures are reprinted with permission from [21]. Copyright 2007, Nature Publishing Group.

Our previous studies suggested that *GALR1* is a tumor suppressor gene [18,20,21]. Also, p27 and p57 are induced, while cyclin D1 is suppressed following ERK1/2 activation [21]. Using *GALR1*-transfected HNSCC cells, we showed that GALR1 signaling inhibits cell proliferation (Figure 1A) and colony formation (Figure 1B), which is associated with ERK1/2 activation (Figure 1C). Consistent with the *in vitro* findings, the tumor formation and growth rates of both Galanin (GAL) and GALR1 expressing HNSCC cells are significantly reduced *in vitro*.

Generally, ERK activation is association with induction of cell proliferation, rather than its inhibition. The mechanism the activated ERK1/2 pathway can induce inhibition of cell proliferation is not completely understood. The ultimate cellular response, such as growth inhibition *versus* cell proliferation, to ERK1/2 signaling would depend on the strength and duration of ERK1/2 activation [22]. For example, transient or lower level ERK1/2 activation may contribute to cell cycle progression, whereas sustained higher levels or prolonged ERK1/2 activation may induce cell growth suppression [22,23]. Small GTP-binding proteins might also play important roles to determine the cellular response to ERK1/2 activation [24]. Indeed, Woods *et al.* demonstrated that lower levels of Ras activation promotes the mitosis of the cells, but higher levels of activation led to increase the expression of p21^Cip1^, which is one of cyclin-dependent kinase inhibitors (CKIs), thereby causing cell cycle arrest [25]. More recently, another Ras family member, Rap1 and B-Raf, a downstream effector of Rap1, have been linked to ERK1/2 activation and consequent cell growth arrest and/or differentiation through a Ras-independent mechanism [24,26]. Our data demonstrate that galanin stimulated ERK1/2 activation increased 15-fold for up to 3 h, and remained above basal levels for 24 h in GALR1-expressing HNSCC cells [21]. Lahlou *et al.* [24] explained that the cellular decision to induce CKIs and cell cycle arrest in G1 phase is determined by the balance of ERK1/2-dependent and -independent mitogenic effects such as PI3K pathway. These findings are consistent with our results, which indicated that galanin and GALR1 induce cell growth suppression though ERK1/2 activation. We also observed that galanin-dependent stimulation of the PI3K is mediated by either GALR2 or GALR3 [21].

**Figure 2:**
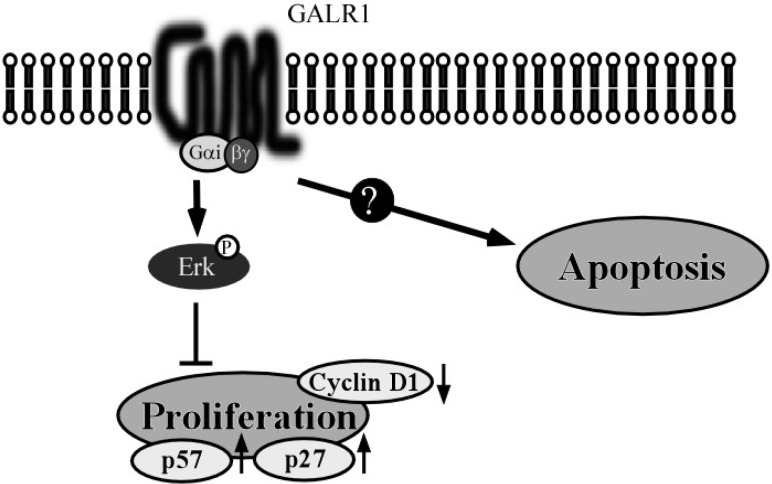
Schema of GALR1 pathway and function in HNSCC cells. In *GALR1*-transduced HNSCC cells, galanin stimulates ERK1/2 activation and suppresses cell proliferation. Galanin stimulation increases expression of the cyclin-dependent kinase inhibitors, p27 and p57, and it also reduces cyclin D1 expression. These signaling pathways are sensitive to pertussis toxin (PTX). GALR1 does not appear to be associated with apoptosis.

The ability by which Gi α-coupled receptors can activate the ERK1/2 pathway is well-known, similar to the Gβγ-dependent pathways that can also activate these kinases. In our study, we observed that galanin and GALR1-mediated ERK1/2 activation was sensitive to PTX, implicating Gi α protein in this signaling cascade. It is well-known that Gβγ subunits also induce ERK1/2 activation by a mechanism involving PI3K pathway [27]. Therefore the contribution of PI3K for GALR1 induced ERK1/2 activation was examined. LY294002, the PI3K inhibitor, did not cancel out either ERK1/2 activation or inhibition of cell proliferation induced by galanin and GALR1 [21]. On the other hand, galanin and GALR1 induced regulation of p27^Kip1^, p57^Kip2^ and cyclin D1 expression and these effects were significantly abrogated by the MEK/ERK inhibitor, U0126 [21]. Thus, GALR1 inhibits proliferation that is required for cell cycle arrest, consequent to ERK1/2 activation though a Giα-dependent pathway (Figure 2).

*p27^Kip1^* and *p57^Kip2^* are defined as tumor suppressor genes. Low p27^Kip1^ expression is associated with poor prognosis in many different tumors, including non-small lung cell carcinoma, gastric carcinoma, and laryngeal carcinoma [28,29,30,31]. High cyclin D1 expression occurs at a high frequency in a variety of carcinomas including those of HNSCC, pancreas, breast and esophagus, and is associated with poor prognosis [32,33]. The fact that GALR1 can down-regulate these cell cycle control genes suggests that it may also exert a tumor suppressor role in HNSCC [21] (Figure 2).

Although Galanin and GALR1 clearly modulate cell growth and proliferation, we did not observe any effect of either protein on other cancer-associated phenotypes such as apoptosis (Figure 2), invasion potential, and mesenchymal–epithelial transition (MET).

### 2.3. Epigenetic Silencing of GALR1 in HNSCC and its Utility as a Prognostic Marker

GALR1 has been investigated as potential prognostic factor in esophageal carcinoma [34], uterine carcinoma [35], and mucoepidermoid carcinoma of the salivary gland [36]. In each case, the correlation between prognosis and methylation of the *GALR1* promoter region was evaluated.

Doufekas *et al.* [35] initially analyzed over 27,000 CpG sites in endometrial cancers and normal endometrial tissue, and then developed a quantitative PCR-based *GALR1* methylation assay to test vaginal swabs from 79 women who had postmenopausal bleeding. They found that methylation of *GALR1* promoter region is one of the most common molecular alterations in endometrial cancer, and it predicted the presence of endometrial malignancy with a specificity of 78.9% and a sensitivity of 92.7% [35].

We hypothesized that *GALR1* would have a tumor suppressor role in HNSCC [21]. In general, tumor suppressor genes may be inactivated by point mutations, homozygous deletions, or loss of heterozygosity and aberrant methylation in intractable cancers. Methylation of CpG sites within the promoter region is often associated with silenced gene expression; within tumor suppressor loci this can engender tumorigenesis. The *GALR1* promoter is TATA-less and contains GC-rich sequences that may be susceptible to DNA methylation and gene silencing [37]. We first determined that the methylation level correlated with degrees to which genes were expressed as revealed by RT-PCR in the HNSCC cell lines. We observed that *GALR1* was partially or fully methylated in 52.7% of HNSCC cell lines, but not in most (90.0%) of the nonmalignant cell lines [38]. Loss of *GALR1* expression is related to hypermethylation of key CpG sites within transcription factor binding domains [38]. In contrast, in cell lines with readily detectable *GALR1* mRNA, CpG sites are only moderately methylated when compared with cells in which the transcript is undetectable [38]. Thus, *GALR1* methylation is significantly correlated with decrease of GALR1 expression. The experiments using clinical HNSCC samples demonstrated that *GALR1* methylation was significantly correlated with reduced survival rates, tumor stage, lymph node status, increased tumor size, cyclin D1 expression and *p16* methylation [38]. In multivariate analysis, taking into account age, tumor site, smoking, tumor stage, and cyclin D1 expression, only *GALR1* methylation and stage were significant predictors of poor survival [38]. These data supported our hypothesis that *GALR1* might be a tumor suppressor gene, and that it could be a potential prognostic factor in HNSCC.

**Figure 3:**
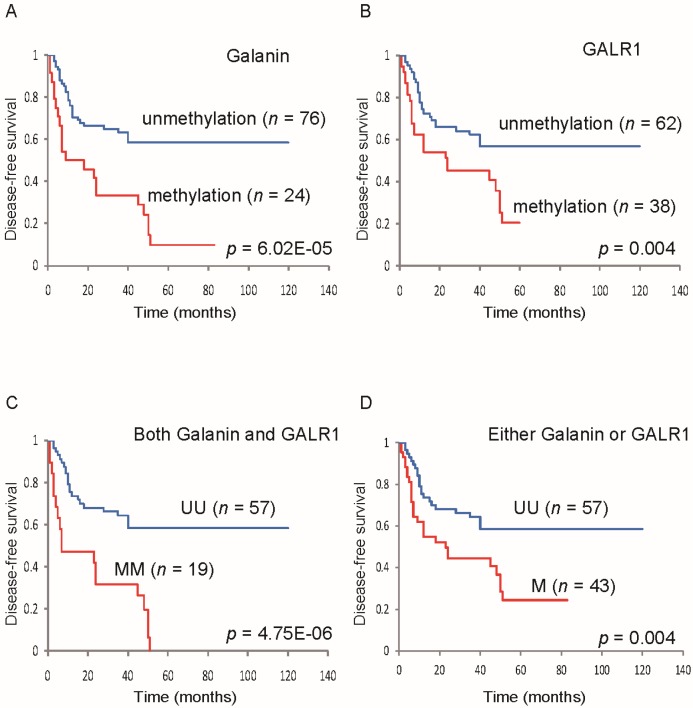
Kaplan-Meier estimates of disease-free survival (DFS) among 100 patients based on their *galanin* and *GALR1* methylation status. The presence of *galanin* promoter methylation was significantly related to a statistically decrease in DFS (**A**); Even *GALR1* methylation alone was significantly related to reduced DFS (**B**); Methylation of both *galanin* and *GALR1* is related to a reduced DFS rate, in comparison to the absence of methylation of both (**C**); Methylation of either *galanin* or *GALR1* was associated with a reduced DFS rate, in comparison to the absence of methylation of either (**D**). Figures are reprinted with permission from [39]. Copyright 2013, Elsevier.

Galanin, which is ligand of GALR1, is also methylated in HNSCC. Indeed, Kaplan-Meier plots showed that *galanin* methylation in clinical tumor samples was significantly related to reduced disease-free survival (DFS; Figure 3A [39]). Patients with *GALR1* methylation also had a significantly reduced DFS (Figure 3B) [39]. Furthermore, methylation of both *galanin* and *GALR1* was associated with a DFS rate of 0%, in comparison to 58.5% in the absence of methylation of both (Figure 3C). Methylation of either *galanin* or *GALR1* was related to a DFS rate of 24.4%, in comparison to 58.5% in the absence of methylation of either (Figure 3D) [39]. The adjusted odds ratio for recurrence when *galanin* was methylated in the primary tumor was 8.95 (*p* = 0.002), and when both galanin and GALR1 were methylated was 23.84. They are significantly higher ratio compared to those who were “methylation-negative” at both loci [39]. These results suggest that monitoring GALR1 and its associated signaling pathways can be used for prognosis in HNSCC.

## 3. Galanin and Galanin Receptor 2 (GALR2)

### 3.1. GALR2 Signaling Pathway

GALR2 signals via multiple classes of G proteins and stimulates diverse intracellular pathways [40]. According to previous reports, the most common pathway of GALR2 involves phospholipase C (PLC) activation, the role of PLC is increase of inositol phosphate hydrolysis, and it mediates the release of Ca^2+^ into the cytoplasm from intracellular stores and opening Ca^2+^-dependent chloride channels [41,42,43]. These intracellular effects by GALR2 are not affected by PTX, and it demonstrates that GALR2 may act though G_q/11_-type G proteins [43]. However, whether GALR2 has functional interactions with other types of G proteins is somewhat controversial. PTX-dependent ERK1/2 activation was observed in *GALR2*-transfected HNSCC cells; however, both PTX and U0126, an ERK-specific inhibitor, partially abrogated GALR2-induced cytotoxicity [44]. Fathi *et al.* observed galanin-dependent cAMP production in HEK-293 cells overexpressing human GALR2 [45]. This effect was PTX-sensitive, which suggests a GALR2 also has Gi pathway that mainly inhibits the cAMP dependent pathway by inhibiting adenylate cyclase activity, similar to GALR1 [43,46]. Other signaling pathways have been proposed for GALR2 though functional coupling to a G_12/13_-protein, the G_q_ phospholipase C/calcium and the G12/Rho pathway. Furthermore, other studies demonstrated that GALR2 is also coupled to a Go-protein that activates MAPK in a PTX-sensitive, PKC-dependent manner [43,47,48]. Thus, GALR2 appears to utilize multiple signaling pathways in order to mediate its effects.

### 3.2. GALR2 Function in HNSCC

**Figure 4:**
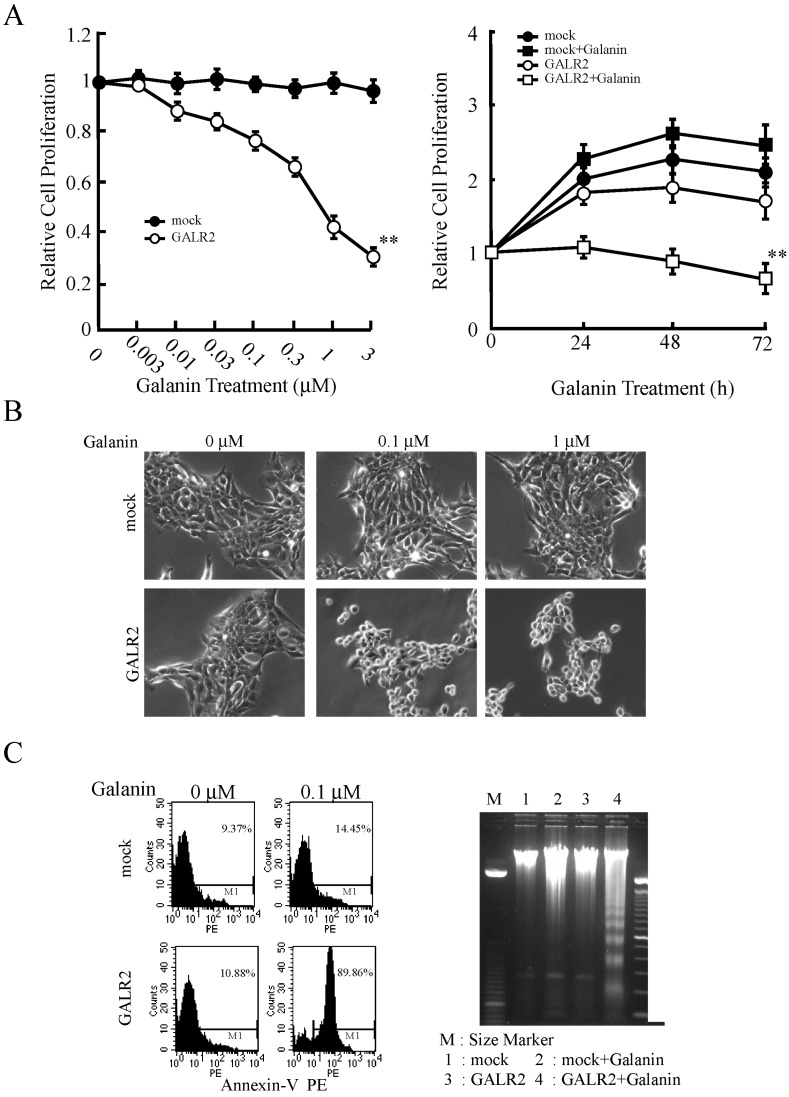
Galanin-induced growth inhibition and cytotoxicity in GALR2-transfected HNSCC cells. (**A**) Proliferation as a function of galanin concentration was measured. Cells were treated with various concentrations of galanin for 24 h (left) and 1 μM galanin for 24 h, 48 h and 72 h (right). Proliferation was significantly inhibited in a concentration- and time-dependent manner (** *p* < 0.01); (**B**) Cell morphology was altered by galanin stimulation in GALR2-transduced HNSCC cells; (**C**) Galanin and GALR2 also induced apoptosis, which was confirmed by flow cytometry for Annexin-V positive cell (left) and analysis of DNA fragmentation using agarose gel electrophoresis (right). Figures are reprinted with permission from [52]. Copyright 2009, American Association for Cancer Research.

As with GALR1, conflicting results were reported on the role of GALR2 in HNSCC. While some studies have shown GALR2 to be proproliferative [49], others indicate that reintroduction of GALR2 into tumor cell lines established from pheochromocytoma, neuroblastoma and HNSCC are susceptible to galanin-mediated apoptosis and/or growth inhibition [50,51,52]. Using cells stably overexpressing GALR2 we also showed that GALR2 has both antiproliferative (Figure 4A,B) and proapoptotic effects (Figure 4C) in *p53* mutant HNSCC cells [44,52,53]. Although these studies demonstrate that GALR2 can induce apoptosis, there are different mechanisms by which GALR2 causes apoptosis.

Berger *et al.* [50] suggested that GALR2-induced apoptosis is caspase-3-dependent. However, the same group showed that a caspase-3 inhibitor was unable to block apoptotic morphology and the inhibition of cell proliferation in galanin-stimulated SY5Y/GALR2 cells. Therefore, they concluded that caspase-3 is not an essential mediator of apoptosis induced by GALR2 activation [50].

Tofigi *et al.* also reported significant caspase activation and morphological changes in GALR2-transfected cells after galanin stimulation [51]. The authors suggested that GALR2 blocks activation of the pro-survival AKT kinase, which leads to a net dephosphorylation of the apoptotic BAD protein and consequent caspase-3-dependent cell death [51]. On the contrary, Sugimoto *et al.* reported synergistic effects on cell proliferation following concomitant upregulation of galanin signaling and downregulation of GALR1 via GALR2 [54]. Banerjee *et al.* demonstrated that GALR2 promoted both survival and proliferation via ERK and AKT signaling cascades in a RAP1-dependent manner in HNSCC cells [55]. They also described in another study that GALR2 induced angiogenesis by secretion of interleukin-6, proangiogenic cytokines and vascular endothelial growth factor via p38-MAPK pathway [56].

Galanin and GALR2 also induced p27^Kip1^, p57^Kip2^ up-regulation and cyclin D1 down-regulation, finally decreased bromodeoxyuridine incorporation [52]. These effects phenocopy the results of GALR1 overexpression in HNSCC.

**Figure 5:**
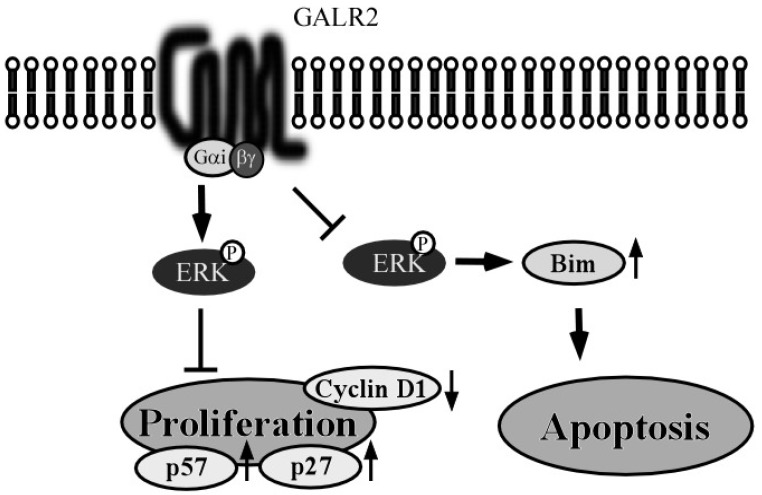
Schema of GALR2 pathway and function in HNSCC cells. In GALR2-transduced HNSCC cells, galanin induced ERK1/2 activation and suppressed cell proliferation. Galanin stimulation reduced cyclin D1 expression and increased expression of the CKIs, p27 and p57. These signaling pathways were sensitive to PTX. Furthermore, a study using AAV vectors revealed that GALR2-mediated apoptosis may also occur in a caspase-independent manner; this involves the induction of the pro-apoptotic BCL2 family member, Bim after downregulation of ERK1/2.

*GALR2* transduced HNSCC cells using adeno-associated virus vectors revealed that it mediates apoptosis in a caspase-independent manner; this likely involves the up-regulation of the pro-apoptotic BCL2 family member, Bim after the downregulation of ERK1/2 [53]. Under these conditions, GALR2 induced cell cycle arrest was not observed; this result is different from previous studies by which the cell cycle arrest was observed following GALR2 activation [52,53], suggesting the difference is due to the different expression levels of GALR2 in the 2 systems. In stably transfected cells, GALR2 activates ERK1/2; this effect is associated with anti-proliferative effects, rather than induction of apoptosis [44]. Thus, the activation of distinct signaling pathways by GALR2 can lead to either ERK1/2 upregulation or downregulation; this differential regulation of ERK1/2 is associated with increased proliferation or activation of apoptosis, respectively. GALR2-dependent signaling pathways and cellular functions are shown in Figure 5. Although the reasons for this discrepancy are unclear, we note that similar paradoxical effects have also been observed in GALR1 signaling. For example, Henson *et al.* reported that the antiproliferative effects by GALR1 activation are due to ERK1/2 inhibition [18], whereas we demonstrated that GALR1 required ERK1/2 activation in order to induce arrest [21]. GPCRs were originally considered to be monomeric membrane proteins, but subsequent studies showed that GPCRs can form both heteromultimers and homomultimers.

In some cases, heteromultimers appear to have specific properties that are not shared with the corresponding homomultimers [57]. However, it is unclear whether this may explain the discrepancies regarding GALR2-induced ERK1/2 activation. This is because there have been few studies that directly address the role of multimeric GALR2 complexes in HNSCC. Further experimental work is thus required to resolve these discrepancies.

In conclusion, while GALR2 activates several signaling pathways, its robust ability to induce apoptosis may be harnessed as part of a therapeutic strategy in the treatment of HNSCC.

### 3.3. Epigenetic Silencing of GALR2 in HNSCC and its Utility as a Prognostic Marker

GALR2 has been investigated as potential prognostic factor in several cancer types. Chung *et al.* reported that *GALR2* hypermethylation indicated a specificity of 95% and sensitivity of 85% in colon cancer from normal tissue, and is also a candidate biomarker for both colon and breast cancer [58]. Yu *et al.* found that GALR2 was among the genes that were hypermethylated in a tumor-specific manner in hepatocellular carcinoma [59]. Furthermore, colorectal cancer patients with GALR2 hypermethylation were more responsive to bevacizumab and cetuximab treatment [60]. These studies suggested that GALR2 is a potential prognostic factor and/or biomarker that can be used to stratify patients prior to treatment.

In our studies of HNSCC, the *GALR1* promoter methylation profile had significant prognostic and biomarker values that could be used for optimal treatment selection [38]. The promoter methylation status of *GALR2* was analyzed in cancer tissues from 36 patients and paired noncancerous mucosae using quantitative methylation-specific PCR [61]. The methylation level of *GALR2* in primary HNSCCs was significantly higher than that in noncancerous mucosal tissues. *GALR2* methylation level also correlated with the degree to which the gene was repressed [61]. The cut-off normal methylation value (NMV, methylated DNA at the target sequence / fully methylated control) for GALR2 was chosen from the receiver operating characteristic (ROC) curve to specificity (100%) and maximize sensitivity (61.1%). In analysis using 100 DNA samples from untreated primary HNSCC tumors, the promoter of *GALR2* was methylated in 31.1% of cases and unmethylated in 69%. Methylation of *GALR2* promoter was significantly related to methylation of *COL1A2*, *H-cadherin*, *DAPK*, *GALR1*, and *Galanin*. Specifically, 38% of the tumors exhibited *GALR1* promoter hypermethylation and 24% of the tumors had Galanin hypermethylation. Eleven percent of the samples from HNSCC tumors were hypermethylated on all three genes of *Galanin*, *GALR1* and *GALR2*, 19% of those tumors were hypermethylated two of three genes, 22% were hypermethylated only a single gene, and 48% were did not methylate any gene [61].

**Figure 6:**
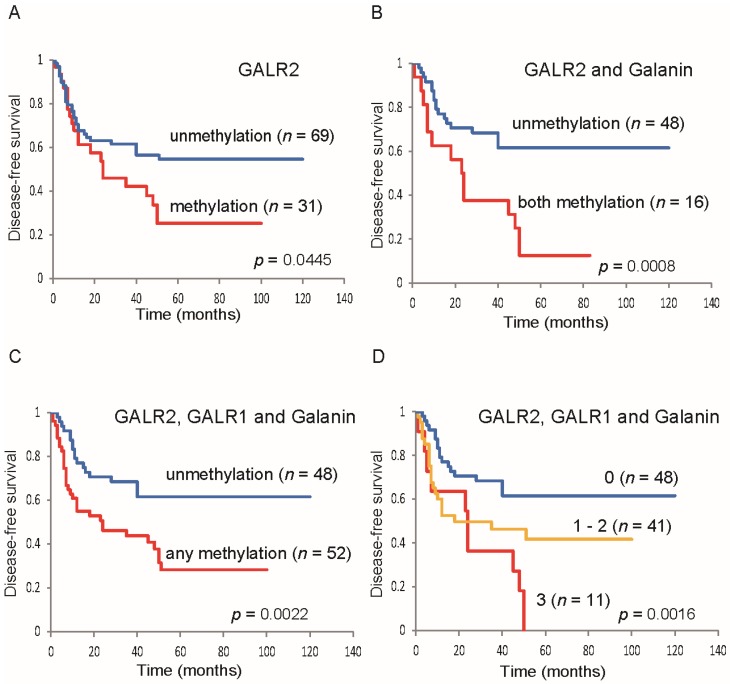
Kaplan-Meier estimates of DFS among 100 patients based on their *galanin* and *GALR2* methylation status. The presence of *GALR2* promoter methylation was related to significant decrease in DFS by a statistical analysis (**A**); DFS of patients with methylation of both *galanin* and *GALR2* was significantly lower than with absence of methylation of these genes (**B**); Methylation of any 3 genes was significantly related to a reduced DFS as compared with the absence of methylation of these genes (**C**); When *GALR2*, *GALR1*, and *galanin* were considered together, the DFS rate of patients with no methylated genes, 1 to 2 methylated genes, and all 3 methylated genes, were 61.6%, 41.7%, and 0% respectively. Differences between the groups were statistically significant (**D**). Figures are from [61]. Copyright © 2013 by John Wiley Sons, Inc. Reprinted by permission of John Wiley & Sons, Inc.

We have also observed that *GALR2* promoter methylation is related to significant decrease in DFS by a statistical analysis (Figure 6A). Methylation of both *Galanin* and *GALR2* was related to a DFS rate of 12.5%, as compared with 61.6% in no methylation of these all genes (Figure 6B). If *GALR2*, *GALR1*, or *Galanin* were methylated, the DFS rate was 28.3%; this contrasts with a DFS of 61.6% in no methylation of these all genes (Figure 6C) [61]. In *GALR2*, *GALR1*, and *Galanin*, the DFS rates of the cases no genes methylated, 1 or 2 genes methylated, and all 3 genes methylated, were 61.6%, 41.7%, and 0%, respectively (Figure 6D) [61]. In a multivariate logistic regression analysis that accounted for sex, age, stage grouping, alcohol intake, smoking status, and methylated genes, the methylation of GALR2 in the primary tumor was related to an adjusted odds ratio for recurrence of 3.12. Both *Galanin* and *GALR2* methylated patients had a significantly higher odds ratio (9.05) for recurrence, compared with those patients in whom neither gene was methylated [61]. Thus, *GALR2* methylation is an independent biomarker in HNSCC, and *GALR2* methylated patients exhibited a high odds ratio for recurrence.

## 4. Tachykinin-1 and Tachykinin Receptor Type 1

The *tachykinin 1* (*TAC1*) gene encodes the neuropeptides, neurokinin A, neurokinin B and substance P; these act through three kinds of transmembrane GPCRs named tachykinin receptors 1–3 (TACR1, TACR2, and TACR3) [62]. Neurokinin A and substance P are alternately spliced products of the *preprotachykinin* gene and are found in the peripheral and central nervous system [63]. Substance P, neurokinin A, and neurokinin B exhibit binding preferences for TACR1, TACR2, and TACR3, respectively [62,64]. These molecules affect motility, the secretion and inflammatory reactions of the gastrointestinal tract though the neurokinin-1 and neurokinin-2 receptors activation [65]. Substance P has proliferative and antiapoptotic effects though activation of the ERK1/2 and nuclear factor-κB pathway [66,67], whereas neurokinin A has antiproliferative properties [68]. TACR1 is expressed in the peripheral and central nervous systems and is indispensable to the maintenance of a favorable tumor microenvironment [69].

When TACR1 is activated by TAC at the plasma membrane, initial G protein-mediated signaling events include activation of phospholipase C (PLC), formation of inositol trisphosphate (IP_3_) and diacylglycerol (DAG); activation of adenylyl cyclase (AC), formation of cAMP, and activation of PKA; activation of phospholipase A_2_ (PLA_2_), formation of arachidonic acid (AA), and generation of PGs, leukotrienes (LX), and thromboxane A_2_ (TXA_2_); and activation of Rock and phosphorylation of myosin regulatory light chain (MLC). Depending on which of these pathways is activated, TACR1 signaling leads to diverse and cell type-specific effects including proliferation, anti-apoptosis, neuronal excitation, inflammation, and migration [70]. These signaling pathways are not significantly different from those that are activated by other GPCRs. However, additional signaling triggered by TACR1 at the endosomal membrane has been reported [70]. This pathway is known as the β-arrestin-mediated endosomal signaling pathway.

**Figure 7:**
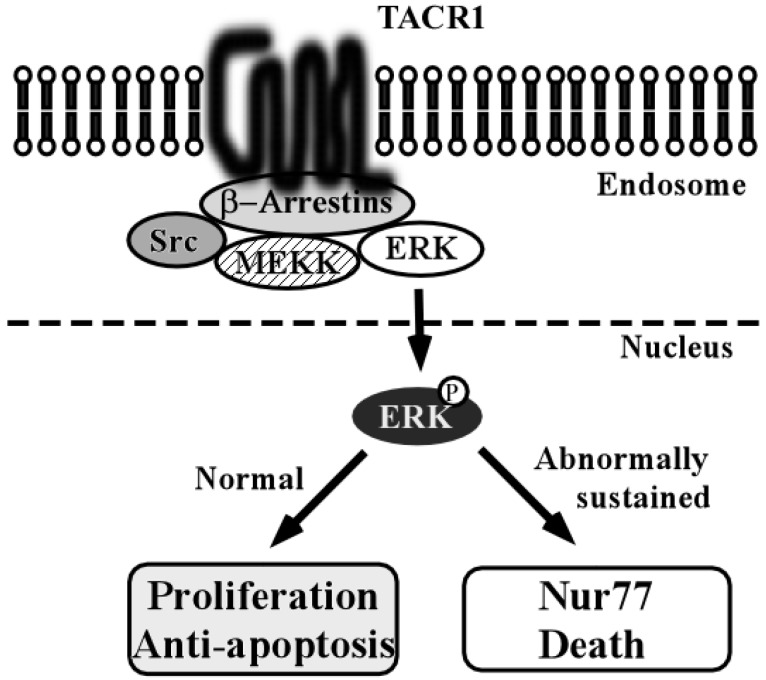
Schema of tachykinin receptor type 1 (TACR1) compartment signaling from endosomal membranes. After β-Arrestin recruits TACR1, Src, MEKK and ERK to endosomes, the complex mediates ERK phosphorylation and activation. β-Arrestin-activated ERK induces both proliferation and Nur77-dependent cell death depending on the cellular context.

After TACR1 activation, β-Arrestin recruits Src, MEKK, and ERK to endosomes and thereby assembles the protein complex that mediates ERK1/2 activation. Under normal circumstances, the activated ERK1/2 translocates to the nucleus and also induces the proliferative and anti-apoptotic action as effect of TAC1. On the other hand, if ERK1/2 activation is abnormally prolonged, as occurs in cells that lack active endothelin-converting enzyme-1, this can lead to phosphorylation and activation of Nur77, which induces cell death (Figure 7) [70]. Although TACR1 signaling pathway status in HNSCC is unclear, this TACR1-induced Nur77 pathway might contribute to the proposed role of TACR1 as a tumor suppressor in HNSCC.

Hypermethylation of *TAC1* was reported in esophageal cancer [71], colon cancer [72], and breast cancer [73]. Overall patient survival is related to *TAC1* methylation status in squamous cell carcinoma, but not in esophageal adenocarcinoma of the esophagus [71]. Despite our understanding of gastrointestinal tract cancer, hypermethylation in HNSCC remains to be explored. To our knowledge, studies of promoter hypermethylation of *TACR1* in human cancer have not been reported. To evaluate the prognostic significance of *TAC* and *TACR1* methylation and their value as biomarkers of recurrence, we examined *TAC* and *TACR1* methylation and related to clinical features in large panels of primary HNSCC specimens [74].

*TAC1* and *TACR1* methylation levels of samples from primary HNSCCs were significantly higher than those from noncancerous mucosal tissues, and correlated with the degree to which mRNA was repressed. The cutoff NMVs for *TAC1* (0.108) and *TACR1* (0.008) were determined by the ROC curves for >95% specificity and high sensitivity [74]. Using this cutoff value, the promoter region of *TAC1* was methylated in 49 of 100 (49.0%) cases, and that of *TACR1* was methylated in 34 of 100 (34%) cases. *TAC1* promoter methylation was significantly related to recurrence events, *p16* methylation, *E-cadherin* methylation, and *galanin* methylation [74].

Kaplan-Meier plots indicated that *TAC1* and *TACR1* promoter methylation in patient tumors were related to the duration of DFS [74]. DFS was related to *TAC1* methylation, but not *TACR1* methylation. Among patients with stage III and IV HNSCC, the 5-year DFS rate in the group of patients with *TACR1* methylation was 31.4%, as compared with 56.7% in the group with nonmethylated *TAC1* [74]. Both *TAC1* and *TACR1* methylation was associated with a DFS rate of 9.8% *versus* 54.9% in neither methylation of them. Both *TAC1* and *galanin* methlation was related to a DFS rate of 0% *versus* 65.9% when both were unmethylated [74]. No significant difference was observed in the DFS of patients with respect to the methylation patters of either *TACR1* or *GALR1*. Multivariate logistic-regression analysis revealed the estimated odds of recurrence related to methylation of *TAC1* and *TACR1*. When *TAC1* methylation was observed in primary tumors, the adjusted odds ratio for recurrence was 3.35 [74]. Patients with both *TAC1* and *TACR1* methylation had a significantly higher an adjusted odds ratio for recurrence, which was 5.09. According to these results, the *TAC1* and *TACR1* promoter methylation profile is an important marker of the clinical outcome following treatment of HNSCC [74].

## 5. Somatostatin and Somatostatin Receptor 1

The main functions of somatostatin (SST) involve inhibition of gastrin-stimulated gastric acid secretion in the gastrointestinal tract, the regulation of endocrine and exocrine secretion, and modulation of motor activity [75]. It has been shown that SST can suppress tumor growth through distinct mechanisms; these include regulation of the immune system, inhibition of growth factors, and reduction in vascularization [76]. Hypermethylation of *SST* has been described in renal cancer [77], colon cancer [72], esophageal cancer [75], and gastric cancer [78], but it remains to be explored in HNSCC.

**Figure 8:**
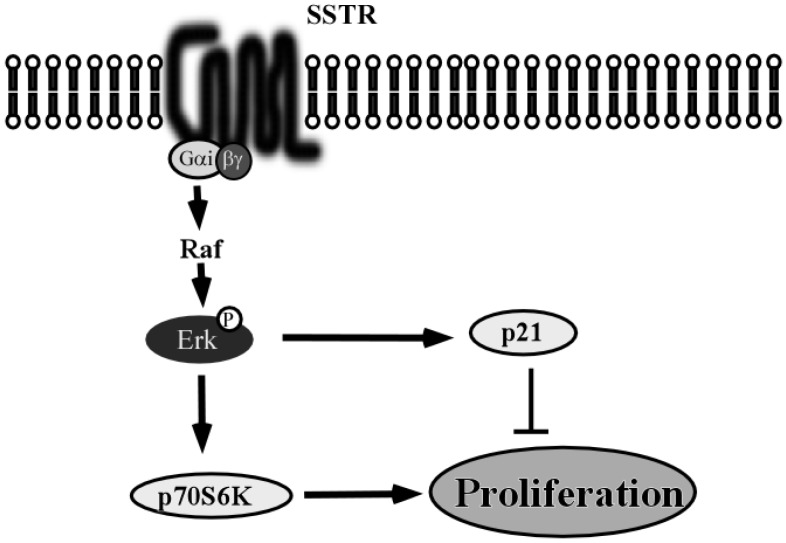
Schema of general SSTR pathway and function. After activation by its ligands, in turn activates Raf, MEK1/2 and ERK1/2. ERK1/2 than activates either p21 or p70S6K, depending on its own level of activation. This leads to, ERK1/2-dependent, p21-mediated cell cycle arrest, or p70S6K-mediated cell growth, respectively [79].

Whether the signaling pathways activated by SSTR in HNSCC are similar to the canonical signaling pathway shown in Figure 8 remains unclear.

In our study, *SST* and *SSTR1* methylation level inversely correlated with the mRNA expression level in HNSCC cell lines. *SST* and *SSTR1* methylation levels in primary HNSCCs were also significantly higher than those in paired noncancerous mucosal tissues, and were associated with highly discriminative ROC curve profiles. Methylation of the *SST* and *SSTR1* promoters was observed in 81 of 100 (81%) cases and in 64 of 100 (64%) cases, respectively. The methylation status of these two promoters was significantly correlated. Methylation of *SST* was significantly related to several clinicopathologic factors, including tumor size, stage, *DAPK* methylation, *TAC1* methylation, and *GALR2* methylation. *SSTR1* methylation was significantly correlated with tumor size, stage, and methylation of *galanin*, *GALR2*, *TAC1*, *TAC1R*, *H-cadherin*, *MGMT*, *DAPK*, and *DCC* methylation [80]. However, the methylation status of *SST* and *SSTR1* of HNSCCs was not associated with any difference in DFS. *SST* and *SSTR1* methylation was not associated with an altered DFS rate when compared with lower methylation levels.

When only patients with oral cavity and oropharynx cancer were analyzed, the DFS rate of patients with both *SST* and *SSTR1* methylation was 48.1%, and that of the other (unmethylated) group was 81.4%. Either *SST* methylation or *SSTR1* methylation elevated the odds of recurrence, but not significantly in multivariate logistic-regression analysis [80].

To investigate the potential value of SST and/or SSTR1 as prognostic factors, we determined the methylation index (MI) [81,82], which for each sample was defined as the number of methylated genes to the number of genes tested (seven in this study; *Galanin*, *GALR1*, *GALR*, *2SST*, *SSTR1*, *TAC1*, and *TACR1*). The DFS was higher in the low MI (0–3) methylated genes group than in the MI (4–7) methylated genes group (64.7% *versus* 14.0%, respectively) [80]. The DFS of patients with both *SSTR1* and *TAC1* methylation was significantly higher than that of patients without methylation. Methylation of both *galanin* and *SSTR1* was associated with lower DFS rate than the absence of methylation (0% *versus* 59.0%, respectively). Patients in whom *GALR2* and *SSTR1* were methylated survived significantly shorter than those in which both genes were not methylated. The DFS of the patients with both *SSTR1* and *GALR1* methylation was significantly higher than that of patients without methylation of these genes [80].

Together, these data indicate that *SST* and *SSTR1* gene inactivation via CpG hypermethylation plays a role during HNSCC tumorigenesis, and that this methylation level may serve as a significant biomarker.

## 6. Future Directions for the Study of GPCRs in HNSCC

GPCRs control various signaling pathways in normal and tumor tissues. More than 30% of all pharmaceuticals’ therapeutic effects are affected by interacting with GPCRs; their importance is underscored by the ever-increasing number of clinical trials associated with modulation of GPCR signaling [11]. The regulation of GPCR signaling in HNSCC has not been examined in a clinical setting. However, we suggest that the study of GPCRs in this disease would contribute to the improvement of HNSCC therapy for the following three reasons.

### 6.1. Loss of GPCR Signaling is a Prognostic Factor in HNSCC

The early identification of patents at high risk for developing distant metastases or local recurrence is critical for the appropriate selection of patients for adjuvant systemic therapy. We hypothesize that specific genetic alternations determine the biological behavior of individual tumors. Such changes can be considered alongside many other candidate prognostic indicators, such as the expression of specific proteins, age, sex, stage, and smoking status. We have focused on the search for genetic makers associated with response to therapy and/or aggressive tumor behavior. Well-known genetic markers for HNSCC are high-risk human papillomavirus (HPV) infection, epidermal growth factor receptor (EGFR) signaling expression and *p53* status [8,83]. High levels of EGFR expression are associated with the undesirable response to chemotherapy/radiotherapy (CRT), induction chemotherapy (IC), and shortened overall survival (OS). High HPV titer is significantly related to high p16 expression, and it was significantly associated with the desirable response to CRT, IC, and OS [83]. Although knowledge related to these genetic markers has led to improvements of therapeutic strategies, the biological behavior of individual tumors is still not fully understood. Accumulated knowledge is therefore required to further improve the response to treatment. Considering the above studies, it appears that the relationship between the reduced expression of specific GPCRs and prognosis may have clinical utility [38,39,61,74].

In the multivariate analysis, *GALR1* methylation and stage were significant predictors of poor survival. Patients with hypermethylated GALR1 had a significantly reduced DFS. Both *galanin* and *GALR1* methylation was associated with a DFS rate of 0%, in comparison to 58.5% in no methylation of these genes [38]. We found that methylation of *GALR2* promoter was also related to significant decrease in DFS [38]. Both *galanin* and *GALR2* methylation was related to a DFS rate of 12.5%, as compared with 61.6% in no methylation of these genes. When considering *GALR2*, *GALR1*, and *galanin*, together the DFS rates for all three methylated genes, 1 to 2 methylated genes and zero methylated genes were 0%, 41.7%, and 61.6%, respectively [38].

*TAC1* methylation in HNSCCs significantly correlated with methylation of *p16*, *E-cadherin*, *galanin*, and reduced DFS. *TAC1* hypermethylated patients in Stage III and IV had significantly shorter survivals than patients without *TAC1* methylation [74]. In multivariate logistic-regression analysis, methylation of either the *TAC1*/*TACR1* gene pair or of *TAC1* was related to an odds ratio for recurrence of 3.35 and 5.09, respectively [74].

Methylation of each specific GPCR is associated with its own discrete value as a prognostic factor. Independently, therefore, each GPCR methylation status has some power for predicting prognosis and/or the response to chemotherapy or radiotherapy. For example, the correlations with both tumor size and clinical stage are similar for several GPCRs with the same methylation status. These clinical parameters are arguably the ones most readily measured. As the number of methylated genes in a given tumor sample increases, so does the predictive power related to both prognosis and/or the success of various treatment regimens.

We suggest that a pressing goal is to establish the global methylation index (GMI), which is the accumulated methylation level of optimal tumor suppressor genes, which can predict the DFS or recurrence rate than the clinical stage and TNM classification.

Recently, various high-throughput technologies founded on bisulfite conversion combined with next generation sequencing (NGS) have been developed and applied to the genome-wide methylation analysis [84]. These types of methods can provide the results of each single base pair, and quantitative DNA methylation level with genome wide coverage. These technological improvements have led to dramatic decreases of the sequencing costs per base, and have greatly accelerated the speed at which high coverage data is obtained [84]. Application of these novel sequencing techniques will greatly facilitate the profiling of GPCR methylation status, and allow accurate attribution of prognostic values for each GPCR locus in HNSCC.

### 6.2. GPCRs as Therapeutic Targets in HNSCC

As more data linking GPCRs to cancer emerge, the pharmacological manipulation of these receptors will become increasingly attractive for the development of novel therapeutic strategies for tumor progression and metastasis. As GPCRs have both oncogenic and tumor suppressive roles, either agonists or antagonists will be required as therapeutic agents, depending on the specific context. Although several clinical trials have already been performed in various cancer types, most have examined the effects of suppression strategies using antagonists, inverse agonists, or antibodies that bind GPCRs. The approach using antagonists or inverse agonists seems particularly attractive, considering the number of compounds that are well-investigated regarding original and adverse reaction, and already approved by regulatory agencies for other indications.

The gonadotropin releasing factor (GnRH) receptor is one such example. Several potent peptide antagonist analogues of GnRH, such as ozarelix, ornirelix, teverelix, LXT-101, iturelix, ganirelix, degarelix, cetrorelix, azaline B, acyline, and abarelix have been clinically investigated. Furthermore, orally delivered non-peptide antagonists are under development for treatment of advanced prostate carcinoma [24].

Endothelin (ET) stimulates the growth of many tumors including breast, lung, ovary, and prostate cancers [6,25]. A phase II trial using ABT-627, an ET-A receptor antagonist, has undergone for treatment of hormone-resistant prostate cancer. Furthermore, chemokine receptors (CXCR), in particular CXCR4, which is the receptor for CXCL12 (SDF-1) were important therapeutic targets in several clinical trials. CXCR4 is also known as a stem cell marker [41] and its importance in cancer progressing is rapidly emerging [10,40,42,43,44]. Ligands inhibiting CXCR4 such as AMD070, AMD3100, AMD3465, BKT140, CTCE-9908, FC131, MSX-122, plerixafor, RCP168, TN14003, T22, and T140 are being evaluated for their efficacy in prevention of metastasis [10,45].

Other than small molecules and peptides as inhibitors, immunological approaches are an alternative means to inhibit the interaction of a GPCR with its endogenous agonist

As therapeutic reagents, antibodies have been raised against the extracellular portion of either the receptors or their ligands. The desired neutralizing effect can be induced by direct injection of antibodies. The purpose of this therapy is to interfere with GPCR signaling between cancer cells the stromal microenvironment, which includes endothelium, myeloid cells, and circulating or local stem cells [3]. Blocking sphingosine-1-phosphate (S1P) with a specific antibody could inhibit endothelial cell migration and capillary formation, and inhibit blood vessel formation caused by reduced the release of IL-6, IL-8, and VEGF from tumor cells [62]. Analogously, proteases that are secreted into the tumor microenvironment respond to protease-activated receptors.

It was already reported that humanized antibodies to CXCL8/IL-8 were shown to inhibit melanoma tumor growth, angiogenesis, and metastasis [64]. Clinical trials that address GPCR and GPCR targeting in HNSCC have not yet been performed. In our opinion, the most promising GPCR signaling pathway to target in HNSCC would be that which involves galanin. Indeed, there are precedents in the literature that targeting galanin signaling in other types of tumors is a valid approach [85,86,87]. As mentioned above, the addition of galanin inhibited the cell proliferation of GALR1-expressing HNSCC cells, though upregulation of ERK1/2 and cyclin dependent kinase inhibitors, whereas in GALR2-expressing cells, the addition of galanin not only suppressed proliferation, but also induced apoptosis [21,52].

Therapeutic targeting of GPCRs in HNSCC is only rational if the identity and levels of specific receptor proteins are known. For this reason, we determined the expression level of GALRs by RT-PCR. Although half of HNSCC patients lose GALR signaling, the other 50% retain intact GALR1 or GALR2 signaling pathways. In these cases, the stimulation of GALR signaling may induce cytotoxic effects in HNSCC cells. The exposure to a GALR2-specific agonist, galanin-like-peptide, induced 2–3-fold more apoptosis compared with galanin in GALR2-expressing HNSCC cells (data not shown). These results suggested that GALRs is potential therapeutic targets of HNSCCs, and development of optimal reagents is required.

Furthermore, there is a close functional relationship between GPCRs and tyrosine kinase receptors. GPCR signaling may precede, follow, parallel, or synergize with signaling activated by receptors that bind platelet derived growth factor and epidermal growth factor (EGF) [11]. As signaling from GPCRs and other receptors converge on several signaling intermediates, the targeting of GPCR signaling may be particularly effective in the treatment of cetuximab-resistant HNSCCs. Indeed, we find that stimulates GALR2-induced apoptosis in cetuximab-resistant HNSCC cells (data not shown). In summary, although targeted therapy based on galanin and GALR signaling is currently lacking in HNSCC, we believe that the above data make a strong case for conducting clinical trials in this area.

### 6.3. Gene Therapy Using GPCRs

Another approach for HNSCC treatment is to exploit gene therapy using virus vectors to restore expression of select GPCRs. Furthermore, HNSCC has several advantages for gene transduction strategies. It is located in the upper aerodigestive tract, meaning that targeted gene transduction can be performed by direct injection of the vector solution. Furthermore, local control would result in significant benefits for patients because metastases mostly occur late in HNSCC progression [88]. Currently, several vectors based on and adeno-associated viruses (AAV), adenoviruses and retroviruses have been utilized for cancer gene therapy. Well-known strategies of gene therapy for HNSCC are immunomodulatory gene therapy, and corrective gene therapy such as adenoviral delivery of *p53* [89].

AAV has a single-stranded DNA and a non-pathogenic virus. AAV vectors have emerged as a useful alternative to other vectors, and have been evaluated in preclinical and clinical models for cystic fibrosis [90], hemophilia [91], and Parkinson’s disease [92]. AAV can also transduce therapeutic gene into HNSCC cells [93,94]. We have transduced HNSCC cells using an AAV vector expressing green GALR2 and fluorescent protein (GFP), and confirmed high GFP expression using a standard vector dose [53]. In the presence of galanin, this vector caused a reduction in cell viability by post-transduction. This appears to involve a caspase-independent form of programmed cell death, although the precise mechanisms await further clarification. Together, these results indicate a bright future for patients with advanced HNSCC.

## 7. Conclusions

Despite increasing of treatment options for patients with HNSCC, survival rates have not improved in the past 30 years. Recent accumulated molecular biological knowledge has facilitated the application of new strategies to improve cancer treatment. Presently, GPCRs are the most studied therapeutic targets in cancer. In this review, we have described four GPCRs that are promising targets for HNSCC treatment. Combined with NGS technology to determine the global methylation indices in biopsies, GPCR-targeted therapy using agonists/antagonists or viral vectors should be explored in preclinical and clinical HNSCC studies. More than one third of pharmaceuticals in the market target less than fifty GPCRs. This leaves hundreds of potential new therapeutic options, including the targeting of more than a hundred orphan GPCRs, as novel opportunities for developing new anticancer agents.
